# Invasive Treatment Options for Gastro-Esophageal Reflux Disease

**DOI:** 10.25122/jml-2020-0160

**Published:** 2020

**Authors:** Vlad Dumitru, Petre Hoara, Daniela Dumitru, Rodica Birla, Cristina Gindea, Silviu Constantinoiu

**Affiliations:** 1. “Carol Davila” University of Medicine and Pharmacy, Bucharest, Romania; 2. General and Esophageal Surgery Clinic, “Sf Maria” Clinical Hospital, Bucharest, Romania

**Keywords:** Invasive, GERD, treatment

## Abstract

Reflux disease continues to be one of the most common pathologies in the world. There is much discussion regarding the mechanism of developing and the variety of possible symptoms. In recent years, the use of new technologies, like high-resolution manometry and pH impedance, brought new insights into this disease. Also, there are emerging therapies that are covering the gap between the patients treated with proton-pump inhibitor (PPI) therapy and those who benefit the most from laparoscopic treatment (hiatal hernia, complications of gastroesophageal reflux disease (GERD). Also, most of them are less invasive than a laparoscopic fundoplication.

We present a short review of the treatment options in patients who need more than lifestyle changes and PPI therapy.

## Introduction

Reflux disease is one of the most common pathologies globally, with a prevalence of up to 31%. It is caused by a multitude of factors, and it also has a wide range of symptoms. The initial diagnosis is clinical, but in some cases, investigations that establish both the definitive diagnosis and the presence of complications must be performed.

The treatment in reflux disease is adapted to the severity of the disease but can also be chosen according to the patient’s preferences. As in other medical fields, the tendency is to tailor the treatment to the patient, but this requires a thorough investigation.

Lifestyle changes (eating habits and others) and weight loss are the first steps. Depending on the symptoms and the underlying pathogenic mechanism, drug treatment with proton pump inhibitors is effective in most cases. However, it is sometimes needed daily for long periods, and it seems to have no effect on the number of reflux episodes in the nocturnal recumbent position [1]. In case of failure of drug treatment, lack of patient compliance, or complications, there are currently more or less invasive therapeutic methods, endoscopic or surgical.

## Material and Methods

Patients with a clinical diagnosis of gastroesophageal reflux disease (GERD), who present any alarm symptoms, or who continue to remain refractory to maximum doses of empirical proton-pump inhibitor (PPI) therapy, should undergo upper endoscopy and other investigations. In recent years, advances in technology permitted the use of impedance-pH monitoring and high-resolution manometry, with the evaluation of the esophagogastric junction (EGJ) function and esophageal peristalsis, for a better characterization of the disease. Drug treatment is the initial choice, PPI being the primary option. Still, some patients require or request a more radical option.

Indications for more invasive treatment include the presence of hiatal hernia or complications of GERD (severe esophagitis or esophageal ulcer, stenosis, bleeding, Barrett’s esophagus), non-compliance with medical treatment, or the patient’s choice.

The beneficial effects of surgery have been clinically proven, predominantly in patients with severe regurgitation, esophagitis, or increased acid exposure of the distal esophagus (quantified by 24-hour esophageal ph monitoring).

Initially was thought that the lack of response to medical treatment is an indication for surgery. However, Morgenthal, Lin and colleagues have shown that patients with GERD symptoms refractory to drug treatment often do not respond to surgical therapy either [2]. The phenomenon is explained primarily by the preoperative failure in making a more rigorous selection of patients in the sense of documenting if the symptoms are clearly related or induced by pathological acid reflux. Particular attention should be paid to distinguish between patients with esophageal hypersensitivity and those with functional heartburn. In the latter, the symptoms are not due to esophageal exposure to reflux.

Although laparoscopic surgery has achieved significant results in treating GERD, studies comparing the effectiveness of drug vs. surgical treatment are quite controversial. Lundell and colleagues showed the superiority of antireflux surgery over omeprazole treatment (20 mg/day) but also found that as the dose increased to 40 or even 60 mg/day, there were no more significant differences between the two in controlling the symptoms [3].

The Society of American Gastrointestinal and Endoscopic Surgeons (SAGES) has established since 2010 the surgical treatment guide for reflux pathology, a document that provides the main surgical indications. Thus, among these, we find: “inadequate control of symptoms, the presence of severe uncontrolled regurgitations, the presence of adverse reactions to drug treatment, lack of compliance with drug treatment, the presence of GERD complications or atypical symptoms” [4].

The main recommendations of the SAGES board also refer to the context in which the surgeon chooses one surgical technique or another. He should be aware that the choice of a fundoplication technique in patients with a poor response to PPI therapy is associated with more inferior results. In contrast, partial fundoplication should be considered as an option in patients with a preoperative diagnosis of “major depression”.

The preoperative objectives, says Moore, should identify the optimal patients for surgical treatment, the results being influenced by the surgical technique [5]. Studies have shown that those with typical symptoms have a better response to the Nissen fundoplication (85% beneficial results at 10 years), while patients with atypical symptoms such as dysphonia, hoarseness, cough, had less satisfactory postoperative results [6]. Those in whom the pathological reflux was identified more frequently in supination have a better response to fundoplication by reducing transient lower esophageal sphincter relaxation (TLESR).

After history-taking and clinical examination, it is important to perform upper digestive endoscopy, which has a specificity of up to 95% in the diagnosis of reflux disease. The technique also allows biopsies to be taken to diagnose and exclude other pathologies that may mimic GERD symptoms: eosinophilic esophagitis, H. Pylori infection, Barett’s esophagus, or adenocarcinoma. If the histopathologic examination reveals a high degree of dysplasia or adenocarcinoma, antireflux surgery is contraindicated.

Other research, such as Chey’s, supports the importance of performing a 24-hr pH monitoring in the context in which upper endoscopy sensitivity is low, more than half of the patients with reflux disease having no macroscopic lesions visible at endoscopy [7]. An increased De Meester score indicates pathological reflux, and the 24-hr pH monitoring can be supplemented with impedance-pH monitoring, the latter being able to differentiate between acid, weakly acid, and non-acid reflux.

Finally, esophageal manometry is useful for finding the precise location of the lower esophageal sphincter (LES), for correct pH probe positioning, in diagnosing associated esophageal motility disorders, and sometimes in guiding the type of surgical approach (total vs. partial fundoplication).

### Predictors of a successful surgical intervention

The SAGES guide includes a series of factors considered “predictors” of the surgical success, the most important in choosing the type of therapeutic intervention being:

• Age - although it may be suspected that the results of antireflux surgery are less satisfactory in adults over 65, experts say that the results are at least in 90% of cases similar to those in young patients, except for the trend of a more extended hospital stay [8].
• Diaphragmatic stressors - a sudden increase in early postoperative intra-abdominal pressure (cough, vomiting, belching, eructation) predisposes the patient to the anatomical failure of the fundoplication;
• Psychiatric pathology - in patients with major depression, more frequent postoperative complications such as severe dysphagia and flatulence have been observed [9].
• Presence of atypical symptoms - patients with asthma, chronic cough, hoarseness, chest pain, recurrent otitis media, dental erosions, idiopathic pulmonary fibrosis, recurrent pneumonia, respond less favorably to surgical treatment [10]. Among these manifestations, it seems that the cough has the best postoperative prognosis with a rate of improvement of symptoms between 69 and 100% [11].
• Motility disorders - in patients with motility disorders, a postoperative risk of increased regurgitation, chest pain, dysphagia was found, especially after performing Nissen fundoplication.
• Reflux pattern - patients with reflux in orthostatism tend to associate pathologies such as aerophagia, which could affect the results of Nissen fundoplication by bloating and increased gas formation [12]. However, some studies describe good or even excellent results of the intervention even in these types of patients, the effect being classified regardless of the position in which the pathological reflux occurs [13].
• Response to preoperative PPI treatment - the poorer the preoperative response to PPI treatment, the less satisfactory are the results [14]. A study conducted over 11 years showed that a lack of response to drug treatment was associated with a success rate of Nissen fundoplication of 56% compared to 77.1% in those who responded to treatment. However, the lack of response to PPI is not part of the category of contraindications to antireflux surgery, studies showing very good results (level II) [15].

Nikolic, Schwameis and Paireder argue that the essential elements in the decision-making process should be functional tests, the severity of the disease (by upper endoscopy), the patient’s history, and history of reflux symptoms [16].

### Laparoscopic surgery

When considered, laparoscopic fundoplication is the first option for more aggressive treatment of GERD. The laparoscopic intervention was associated with a lower rate of intra- and postoperative complications such as esophageal and gastric perforation (0-4%), pneumothorax (0-1.5%), or pleural lesions, when compared to the open approach, depending on the technique and surgeon’s experience [4]. The latter is reserved for complicated cases, with previous upper left quadrant surgery or the need for conversion.

Compared to classical surgery, in 12 clinical trials, laparoscopic surgery was associated with a longer operating time (40 minutes on average, depending on the learning curve), with a hospitalization period of less than 3 days [17], a resumption of daily activities after only one week (level I) and also with two times lower rate of reinterventions [18].

### Nissen, Toupet, or Dor?

Nissen fundoplication (NF) has been considered since the second half of the twentieth century as the “queen of antireflux surgery”, undergoing changes over time to reduce side effects. The 360 ° fundoplication was modified to 270° (Toupet) or even 120° (Dor), the last two being associated with a lower degree of postoperative dysphagia [19].

In systematic reviews and meta-analyzes concerning the type of surgical approach to reflux disease, the Toupet technique showed a rate of 8.5% of postoperative dysphagia compared to 13.5% in Nissen [20].

Randomized studies comparing the effects of anterior/120°/Dor fundoplication with Nissen over a period of 10 years claim that Dor, although associated with a lower degree of postoperative dysphagia, is less effective in controlling pathological reflux over a longer period [21].

Another study comparing short-term and long-term results after the three types of fundoplication stated some conclusions: Nissen has the benefits of good long-term efficacy in controlling the symptoms, with the disadvantages of increased postoperative dysphagia, bloating, and flatulence [22]. The Toupet posterior fundoplication shows reduced postoperative dysphagia and good control of reflux symptoms but requires a longer valve for better results [23]. The Dor (anterior) fundoplication is associated with recurrent symptoms and sometimes requires reintervention for better reflux control.

Analyzing the clinical results of antireflux surgery, Contini and Scarpignato observed that one of the parameters that best quantifies the “treatment success” remains the patient’s satisfaction after surgery [24].

Neither symptomatology (typical/atypical) nor abnormal results on the 24-hour pH monitoring can help establish predictions of surgery response. Instead, the researchers say, a positive preoperative value of the Symptom Index (>50%) observed during 24-hour pH monitoring is a key element in assessing the treatment’s success.

### Other minimally invasive treatment options

Although Nissen fundoplication remains the gold standard in the surgical treatment, some new minimally invasive techniques such as magnetic sphincter augmentation (MSA), electrical stimulation (ES) of LES, endoscopic mucosal resection, or fundoplication have recently been promoted.

Thus, studies on the use of electrical stimulation of LES demonstrate a reduction in symptoms, leading to decreased pathological acid exposure [25]. However, it appears that patients have a higher rate of dysphagia post-MSA compared to Nissen fundoplication or electrostimulation (24% vs. 16% vs. 0%, p <0.04).

### Injection and implantation techniques

In the mid-2000s, a series of LES injection techniques were launched on the market for various inert polymers such as Polymethylmethacrylate (PMMA) in order to provide mechanical sphincter support, decreasing the transient relaxations of LES.

Plexiglas, Durasphere, Enteryx, and Aluvra are just a few of them, the latter still being tested. Created for mild/moderate forms of GERD, they use a concept developed since the 1980s when the use of polytetrafluoroethylene (Teflon paste) in combination with bovine collagen has resulted in accelerating gastric emptying and reducing esophagitis [26].

In a pilot study, Ganz and colleagues demonstrated on a small group of patients (n = 10) the effectiveness of Durasphere treatment at 1 year after the intervention. The need for antiacids has been reduced by more than 50% in 70% of patients, 30% of them giving up completely the PPI treatment [27].

In the case of Plexiglas and Durasphere, there was a problem of durability, the effectiveness decreasing after 6 months, leading to the withdrawal of the products from the profile market [28].

### LINX®

Using the saying “Restore, do not reconstruct” [29], the antireflux device launched in 2008 by Ethicon, US, with the size of a 25 cent coin, uses a ring made of a string of titanium beads with a magnetic effect. The device is implanted laparoscopically at the gastroesophageal junction, assuming that the beads that make up the ring will increase the LES pressure by magnetic attraction.

The main indications are patients with uncomplicated but confirmed gastroesophageal reflux (on pH monitoring), with a body mass index (BMI) <35, no/small hiatal hernia, and normal esophageal motility.

The main contraindications involve patients allergic to titanium, stainless steel, nickel, or other ferrous materials. There is also a lack of clinical trials regarding using the method for patients with hiatal hernia over 3 cm, Barett’s esophagus, esophagitis grade C/D, implantable devices, or esophageal motility disorders [30].

### ENDOSTIM

EndoStim is an implantable neuromodulator that releases electrical stimuli at the LES level that is made of three components: a bipolar stimulator, an implantable generator, and a data analysis software [31]. The electrode is implanted laparoscopically in the LES, and the generator is placed under the skin in a “pocket” located in the upper left quadrant of the abdomen.

It is assumed that acid reflux is controlled by electrical stimulation (controlled by the external program that communicates in a wireless manner with the implantable pulse generator (IPG) by altering the relaxation pressure of the LES.

However, it is recommended that the device should be used only in medical centers highly experienced in the treatment of reflux pathology [32].

### Stretta®

Since its launch on the market, more than 200,000 patients have benefited from this procedure, with more or less contested results. The device uses pulsed radiofrequency (low-frequency energy) waves to reshape the gastroesophageal junction and LES, acting equally on the vagal fibers at the cardio-esophageal level.

The SAGES treatment guide included Stretta as a therapeutic option for GERD in older patients with typical symptoms (heartburn and/or acid regurgitation) for at least 6 months who have partially responded to or are refractory to drug therapy or who have refused fundoplication.

Regarding the technique, patients are first prepared for upper endoscopy under sedation. The role of endoscopy is to measure the distance between the incisors and the Z line. Following that, after removing the endoscope, the radiofrequency (RF) electrode (consisting of a flexible basket-balloon assembly with four needle electrode sheaths) will be inserted using a guidewire and positioned 1 cm above the Z line depending on the distance previously established. The 4 needle electrodes are positioned at a preset length of 5.5 mm on which occasion the RF release is initiated. Each electrode emits waves for 60 seconds, long enough to reach the target temperature of 85°C. The electrodes are subsequently removed, and endoscopy is repeated [33].

A meta-analysis of 1441 patients treated with this procedure concluded its effectiveness in reducing symptoms by significantly decreasing acid exposure, without effects on the normalization of pH. However, some studies are supporting the short-term effect[34].

The trials showed good tolerability of the method; out of 2774 patients, only 5 presented severe complications: 3 esophageal perforations and 2 deaths from aspiration pneumonia [35]. Minor complications include fever, superficial mucosal lesions, or severe chest pain that required opioids.

Suyu, Fei and colleagues, in a comparative study on drug treatment (PPI) vs. Stretta, showed that after 6 months, good results were obtained in controlling the dose and improving the quality of life in both groups of patients. However, those undergoing the Stretta procedure showed a higher degree of satisfaction (80% vs. 30%), 60% of them completely giving up postoperative PPI therapy [36].

The recommendation is that the Stretta procedure should be used primarily for patients with non-erosive reflux disease (NERD), the lack of erosions indicating a lower risk of complications.

A comparative study between the role of Stretta and Toupet fundoplication in controlling the extraesophageal symptomatology highlighted the importance of both procedures; however, the latter is considered superior [37].

### Transoral incisionless fundoplication (TIF)

It is one of the most widely used methods of endoluminal restoration of the Hiss angle and gastroesophageal junction high-pressure zone. The procedure is performed using an EsophyX device, from 2016 with EsophyX Z (EndoGastric Solutions, Inc. Redmond, WA, USA), generating a fundoplication of 270° by apposition of the gastric fundus to the distal segment of the esophagus and fixing it with polypropylene staples [38].

The 2015 RESPECT study included patients with symptoms defined as “problematic” (acid regurgitation and heartburn), dependent on a daily dose of PPI (omeprazole 40 mg or equivalent) for more than 6 months, without motility disorders detected on manometry [39]. TIF improved symptoms in 67% of cases, compared to 45% of cases treated with medication, reducing acid exposure without normalizing ph monitoring results.

In 2018, the TEMPO study reiterated the hypothesis developed by the RESPECT study, mentioning that it evaluates the effect of the two methods over time [40]. The authors observed the disappearance of regurgitations in 88% of patients at 1 year, at 90% at 3 years, and 86% at 5 years. As for the remission of atypical symptoms, they were eliminated in 82% of patients at 1 year, reaching up to 80% at 5 years, without the occurrence of serious complications. After 5 years from the procedure, only 34% needed a daily dose of PPI.

Regarding the complications of TIF using EsophyX, 3 to 10% of patients had bleeding (3-5% required transfusions), endoscopic perforation, pneumothorax, or mediastinal abscesses (less than 2% of cases), the clinical selection of patients being the key to successful therapy [41].

### GERD X

The GERD X (G-SURG GmbH, Seeon-Seebruck, Germany) is an endoscopic technique that is performed under general anesthesia. A Savary guide wire is inserted at the gastric level by means of a gastroscope, following which the distal end of the GERD X system will be inserted and retrofitted towards the anterior part of the cardia (approximately 1 cm below the junction). A thin endoscope will be inserted through a specially designed channel of the device, with the help of which the gastroesophageal junction will be visualized. The GERD X arms open, and an endoscopic tissue retractor is advanced deep into the cardia. The retractor is pulled to tighten the tissue between the open arms of the device, followed by a subsequent tightening of the arms to make a transmural suture (one or two until the tightening of the gastroesophageal junction around the endoscope).

At the end of the procedure, the GERD X device and the gastroscope are removed, with the subsequent reintroduction of the gastroscope to evaluate the quality of the application.

The technique is well-tolerated, improves symptoms, quality of life, 60% of patients showing a normalization of pH (quantified by the De Meester score) [42]. Only 10% of patients required PPI daily, 26.7% on-demand, without significant effects on manometric characteristics in the context in which the technique does not involve structural changes in the esophageal hiatus. Koch, Witzel and Weitzen recommend the method as a good alternative to the chronic use of PPI by reducing the exposure of the distal esophagus to acid and improving typical symptoms related to reflux and quality of life [43].

### Medigus Ultrasonic Surgical Endostapler (MUSE)

Another type of endoscopic fundoplication is MUSE. Compared to other fundoplication devices, the device has its own camera, light source and an ultrasound transducer, being able to visualize the procedure directly and assess the thickness of the tissue involved.

It has comparable results with other endoscopic techniques; at the 4-year follow-up, 69.4% of patients were not taking PPIs daily anymore [44, 45].

### Anti-reflux mucosectomy

In cases of GERD, with no/smaller (<2cm) associated hiatal hernia, one endoscopic treatment option is the circumferential resection of mucosa around the gastroesophageal junction, at 180-270°. During the healing process, a narrowing of the space is leading to a decrease in reflux symptoms. The first procedure was done in order to treat Barrett’s esophagus, but the additional positive effect on GERD was then observed [46]. Since then, a number of publications showed, in a limited number of cases, good results in controlling reflux symptoms, although with some side effects, like stenosis requiring balloon dilation [47].

## Conclusions

Although the surgical approach of patients with reflux disease refractory to drug treatment is considered the optimal solution, preoperative evaluation plays a critical role in maximizing postoperative outcomes.

Age, factors that increase intra-abdominal pressure, psychiatric pathologies, atypical symptoms, reflux pattern, motility disorders, or response to preoperative PPI treatment are described as some of the most important factors that dictate the choice of a specific surgical technique over others.

## Conflict of Interest

The authors confirm that there are no conflicts of interest.
